# The epistemic innocence of clinical memory distortions

**DOI:** 10.1111/mila.12175

**Published:** 2018-02-20

**Authors:** Lisa Bortolotti, Ema Sullivan‐Bissett

**Affiliations:** ^1^ Department of Philosophy University of Birmingham Edgbaston UK

**Keywords:** dementia, distorted memory, epistemic benefits, epistemic innocence, psychological benefits, well‐being

## Abstract

In some neuropsychological disorders, distorted reports seem to fill gaps in people's memory of their past, where people's self‐image, history, and prospects are often enhanced. False beliefs about the past compromise both people's capacity to construct a reliable autobiography and their trustworthiness as communicators. However, such beliefs contribute to people's sense of competence and self‐confidence, increasing psychological well‐being. Here, we consider both the psychological benefits and epistemic costs and argue that distorting the past is likely to also have *epistemic* benefits that cannot be obtained otherwise, such as enabling people to exchange information, receive feedback, and retain key beliefs about themselves.

## 1 INTRODUCTION

Some neuropsychological disorders cause people to experience serious memory impairments. Often, people report distorted memories and confabulate in those contexts. One example is Alzheimer's disease (see, for instance, MacDuffie, Atkins, Flegal, Clark & Reuter‐Lorenz, 2012, p. 509). In this article, we are interested in those clinical memory distortions (hereafter CMDs) that are common in people affected by dementia. False beliefs about the past, present, and future compromise people's understanding of their physical and social environment, their capacity to construct a reliable autobiography, and their trustworthiness as communicators. But by filling gaps in the memory of their past with inaccurate details, often enhancing their self‐image, personal history, and future prospects, people gain self‐confidence and a sense of competence, ultimately increasing psychological well‐being. Our thesis is that CMDs have psychological benefits that come at an epistemic cost in some contexts but are also likely to have epistemic benefits, such as increasing people's chances of exchanging information, obtaining feedback from peers, and enabling people to retain some key beliefs about themselves notwithstanding their significant loss of autobiographical memory.

In Section 2, we outline the context in which a discussion of the psychological and epistemic effects of CMDs is relevant. In Section 3, we describe the epistemic costs of CMDs, and in Section 4, we review some of their psychological benefits. In Section 5, we introduce a framework, that of *epistemic innocence*, with which to make sense of the idea that some cognitions can be both epistemically costly and epistemically beneficial at the same time. We then argue that, in some contexts, having CMDs satisfies the conditions for epistemic innocence. In Section 6, we consider some of the practical implications of our thesis.

## 2 A TRAGIC CHOICE

The idea that well‐being is sometimes promoted at the expense of knowledge is not new in psychology (e.g., Taylor, 1989). In the context of distorted memories about the past, the idea has been discussed in relation to SPECAL, a method of caring for people with Alzheimer's disease, explained and defended in Oliver James's book *Contented Dementia*. Some of the features of the SPECAL method are very controversial, such as the idea that we should not contradict people with Alzheimer's disease when they report something false and that we should make them relive situations that do not apply to them anymore (‘make a present of the past’) if such situations make them feel good and confident. The recommendations of the SPECAL method have been interpreted as an invitation to tolerate, and sometimes actively encourage, false representations of reality. Here is what the Alzheimer's Society UK says about the SPECAL method:

We do fully support the need to understand the perspective of the person with dementia and to provide an environment which meets social and psychological needs. However, we struggle to see how the systematic deception of SPECAL, however well intentioned, is in the best interests of the person with dementia. Individualised care requires appreciating the relevance of someone's unique history for the present, not encouraging the person to continually 'live in the past'. (Alzheimer's Society, 2012)

In the debate, both supporters and critics of the SPECAL method view its alleged psychological benefits as a by‐product of carers not challenging or actively encouraging distortions of reality. There seems to be a tragic choice: people can either be detached from reality but happy or in touch with reality but miserable. If CMDs are not challenged, no genuine feedback is provided, and the person will continue to have false beliefs about the past but will feel competent and confident. If CMDs are challenged, the feedback may lead the person to reject some false beliefs about the past but will also cause the person to doubt her competence, giving rise to anxiety and social withdrawal.

In this article, we suggest that there ought to be more to the debate on how to manage CMDs than this tragic choice. First, whereas some of the psychological gains attributed to CMDs depend on the memory presenting an enhanced self, other psychological gains do not depend on having a distorted memory rather than a non‐distorted one but on having a distorted memory rather than having *no memory at all*. Thus, it is important to think about whether there is an alternative to having a distorted memory that is available to people with Alzheimer's disease.

Second, the fact that increasing well‐being has positive epistemic consequences means that, in some contexts, having a distorted memory can be good not just from a psychological point of view but also from an epistemic one. Thus, it is important to think about the overall epistemic effects of CMDs and not focus exclusively on the downsides. This does not commit us to the implausible claim that the epistemic benefits of CMDs *outweigh* their epistemic costs. Rather, it commits us to viewing CMDs as a means to averting a breakdown in epistemic functionality at a critical time (as we shall explain in Section 6).

There is a framework that can be applied to cognitions that are at the same time epistemically costly and epistemically beneficial, enabling us to think about the available alternatives to, and the epistemic benefits of, having a CMD. This is the framework of *epistemic innocence*: in at least some contexts, an epistemically costly cognition can also have some significant epistemic benefit that could not be attained by less epistemically costly means. Such cognitions can be described as “epistemically innocent”, and examples include unrealistically optimistic beliefs (Bortolotti, Antrobus & Sullivan‐Bissett, forthcoming), delusions (Bortolotti, 2015, 2016), confabulatory explanations (Sullivan‐Bissett, 2015), psychedelic states (Letheby, 2016), and inaccurate social cognitions (Puddifoot, 2017). We will argue that some CMDs are also epistemically innocent.

## 3 EPISTEMIC COSTS OF CLINICAL MEMORY DISTORTIONS

When we talk about CMDs, we refer to those reports of past autobiographical events by people who (a) have a clinically relevant memory impairment; (b) misrepresent reality in some important respect; (c) have no awareness of the misrepresentation (Talland, 1965); and (d) have no intention to deceive (Moscovitch, 1995). We are especially interested in the status of memory reports that contain some true information about the past and are not completely fabricated or implanted (Gallo, 2010; Loftus, 1996; Mendez & Fras, 2011). We take CMDs to include both distorted memories and instances of confabulation (Van Damme & d'Ydewalle, 2010, p. 212) that are elicited by questioning and whose content can be plausible and may or may not be bizarre (Kopelman, 1999, pp. 197–198; Hirstein, 2005, p. 20).

The distortion of a past event can be *motivationally neutral* when the information that has been lost is replaced with plausible but inaccurate information, independent of whether the inaccurate feature of the event is pleasant or unpleasant. In other cases, the distortion can be construed as *motivationally biased*, and the distortion can be seen as part of a defence mechanism. Defence mechanisms are defined as ‘means of nuancing or processing information such that it is rendered less anxiety‐provoking’ (McKay, Langdon & Coltheart, 2005, p. 316). The person enhances the past by denying something unpleasant or providing an embellishment or a rationalisation of it (Flament, 1957).

CMDs are often characterised on the basis of their epistemic costs: the content of the distorted or confabulated report does not match reality, is typically more vague and abstract than that of accurate reports, and may be inconsistent with other things the person believes or remembers (see Addis & Tippett, 2008; Conway, 2005; Kopelman, 1999). When people form a personal narrative encompassing their experiences, the inclusion of memory distortions may lead to a lack of *correspondence* between the narrative and reality and a lack of internal *coherence* in the narrative. Furthermore, CMDs that are recognised as such by others may function as an obstacle to fruitful social interactions. The person doing the reporting can be sanctioned for the distortion and excluded from exchanges of information with peers.

Memory distortions are often due to two common ‘sins of memory’, misattribution, and bias (Schacter, 1999). In misattribution, people remember an event, but key features of the event are misrepresented, such as when the event happened or how people learned about it. In bias, people's existing beliefs, feelings, and opinions affect the way a past event is remembered. In the context of dementia and other neuropsychological disorders, reports of past events are vulnerable to both misattribution and bias. One instance of misattribution is when salient life events are not reported in chronological order or may be ignored altogether (e.g., the death of a loved one is forgotten). In this case, the person's self‐narrative becomes less accurate and more fragmented as systematic connections among life events are less straightforwardly identified.

Instances of bias are also frequently observed in CMDs, such as an enhanced description of oneself or one's relational life. People with neuropsychological disorders may describe themselves as healthier or more independent than they actually are, as if they were still engaged in professional activities as opposed to being cared for at home or in hospital. They may also exaggerate what their previous social or financial status was (Fotopoulou, 2008, pp. 550–551). Conway and Tacchi (1996) report the case of a woman with amnesia, OP, who ‘rewrote’ the story of her family relationships to emphasise love and cohesion and downplay tension and disagreement. Such a departure from reality may be detrimental in several respects. The person who reports motivationally biased memories can be perceived as an unreliable communicator and challenged when her reports appear as implausible to her peers. As a result, the person may refrain from further exchanges for fear of sanction and disapproval, and this rules out the possibility of feedback and cross‐checking.

Memory reports may create the illusion of greater continuity between the person‐prior‐to‐the‐onset‐of‐the‐illness and the person‐experiencing‐memory‐loss. The person attributes to herself capacities, concerns, and personality traits she no longer has because the self‐narrative does not update successfully when circumstances change. When memories of misrepresented events are woven into the person's self‐narrative, they are likely to generate tension with other beliefs and memories and give rise to further distortions and confabulations (so‐called ‘secondary confabulations’). These further confabulatory explanations are developed to account for the tension between existing cognitions, although the person does not realise this. As a result of striving for coherence, the self‐narrative becomes more implausible, and this can negatively impact the capacity to make good decisions and accurate predictions about the future (Suddendorf, Addis & Corballis, 2009, p. 1320).

To sum up, we offered some reasons in favour of what we will call the *Epistemic Costs claim*: CMDs are epistemically costly as they misrepresent reality, sometimes by embellishing it. This results in people being inaccurate or incoherent autobiographers and untrustworthy communicators.

## 4 PSYCHOLOGICAL BENEFITS OF CLINICAL MEMORY DISTORTIONS

Despite the negative aspects of CMDs, the empirical literature suggests that reporting the past inaccurately may have some psychological benefits. We shall look at two such benefits here. Distorted reports of autobiographical events by people with severe memory impairments may increase people's self‐confidence by providing an illusory sense of competence and may increase people's sense of self‐worth by enabling the construction of a better self and of a better reality.

### 4.1 Illusion of competence

CMDs can offer an illusion of competence. That is because CMDs are associated with an increased need for completion and integration (Mendez & Fras, 2011). People with some neuropsychological disorders tend to make inaccurate reports about their past because they have a gap in memory that they feel a strong inclination to fill. The gap‐filling aspect of memory distortions is a potential psychological benefit that prevents *dumbfounding* (Kopelman, 2010), that is, when people are lost for words and cannot answer questions or explain events. By providing an inaccurate report, the person avoids saying ‘I don't know’ (Hirstein, 2005, p. 30; Van Damme & d'Ydewalle, 2010, p. 220).

Another interesting phenomenon is that of *secondary confabulation*, when people provide answers to reconcile a previously made false report with apparently conflicting information, further distancing themselves from reality. Such confabulations often involve a false account of past events and can also support a sense of competence that turns out to be illusory. Here is an example (Moscovitch, 1995, p. 228). A man with widespread frontal damage (HW) correctly reports that he is married and that he has four children, but when he is asked how long he has been married, he replies incorrectly ‘4 months’ instead of ‘30 years’. HW develops links between his current experiences and the past experiences he remembers in a superficial and fragmented way. When he is made aware of the inconsistency in his answers, he confabulates further, reporting (incorrectly) that his children were adopted.

Q. How did you get these children in four months?

A. They are adopted.

This confabulation maintains consistency between HW's claims that he is married with four children and that he has been married for just 4 months.^1^ We find a similar phenomenon in clinical delusions and hypnotic analogues of clinical delusions (Bortolotti, Cox & Barnier, 2012), where people answer a question aimed at challenging their delusional beliefs. In such circumstances, people tend to offer a confabulatory explanation involving a distorted account of the past. The incorrect report is a response to a challenge that helps the person preserve a sense of competence and coherence.

### 4.2 Positively biased identity

In some influential contributions to the psychological and philosophical literature on memory and the self (Goldie, 2012; Hydén & Örulv, 2009; Schechtman, 1996, 2007), ‘sense of self’ or ‘identity’ refers to beliefs about oneself answering two basic questions: (a) *Which* person am I? and (b) *What type of* person am I? Thus, the sense of self comprises both beliefs about one's life story (e.g., when one was born and who one's parents and siblings are) and beliefs about one's personality traits and psychological dispositions (e.g., whether one has a generous disposition and is good at playing football).

Memory is central to the creation and preservation of a sense of self. For instance, Schechtman (1994) argues that autobiographical memories provide a sense that the self is persisting and play an essential role in the construction of personal narratives. Impaired autobiographical memory compromises the capacity to maintain and update one's sense of self. Identity is defined on the basis of *continuity*, that is, seeing oneself as extended in the past and the future, and *distinctiveness*, that is, seeing oneself as different from others (Spini & Jopp, 2014). Memory is instrumental to preserving continuity and distinctiveness, so when memory is threatened, identity is too.

When autobiographical memory is impaired, the sense of self is typically weakened because people struggle to form and report beliefs about their life stories or their lasting features. For instance, as dementia progresses, one's understanding of one's profession, family role, leisure activities, and personal attributes decreases (Cohen‐Mansfield, Golander & Arnheim, 2000). The weakening of the sense of self in the context of neuropsychological disorders featuring memory impairments is correlated with reduced well‐being, and interventions aimed at enhancing identity have been found to contribute to well‐being (Caddell & Clare, 2012). For instance, activities incorporating information about the self increase well‐being and engagement and decrease agitated behaviour in people with dementia with respect to activities that do not incorporate such information (Cohen‐Mansfield, Parpura‐Gill & Golander, 2006).

On the basis of these findings, we would expect that the weaker the sense of self, the steeper the decline of well‐being. However, people in the *advanced* stages of dementia who have a more severe memory impairment experience *increased* well‐being with respect to those who only have a mild memory impairment (Jetten, Haslam, Pugliese, Tonks & Haslam, 2010, p. 414). Why? We know that older people tend to pay more attention to positive stimuli, and this applies especially to autobiographical information. ‘Positivity biases’ enhance well‐being, and this partly explains why the older (non‐clinical) population reports an increase in positive affect in spite of cognitive decline (Mather & Carstensen, 2005). Only positive self‐related past events are remembered. One hypothesis is that in the advanced stages of dementia, there are fewer reality constraints operating on memory, and people enhance their own image, more radically reconstructing their past selves more freely (Jetten et al., 2010, p. 415).

The construction of a *radically enhanced* self is also common in people with neuropsychological deficits who build a new identity for themselves by confabulating. They can present themselves with more skills, more impressive achievements, a profession with higher status, or improved family circumstances than is the case (Fotopoulou, 2008, p. 548). One example is RM, a person confabulating after brain damage caused by a car accident:

While many of RM's confabulations referred to true past events, these tended to be highly exaggerated in ways that enhanced RM's abilities and achievements. For example, while RM had indeed been a good soccer player at school and had been once named ‘player of the year’, RM often referred to this event as having happened 4 or 5 years in a row. (Fotopoulou, 2008, p. 555)

According to Aikaterini Fotopoulou, confabulations enhancing the person's past experiences have positive effects on mood regulation, and in some contexts, they have clinical advantages too. Thus, this is a good example of a psychological benefit that can also be adaptive in other ways.

Self‐related information can be enhanced in memory distortions by people ignoring something unpleasant about themselves. For instance, in the context of dementia, people often undergo a change in their personality traits as a result of the illness. In particular, an increase in *aloof/introvert* and *unassured/submissive* personality traits is observed. People may have no awareness of these changes and tend to describe themselves as they were before the illness (Rankin, Baldwin, Pace‐Savitsky, Kramer & Miller, 2005). People with frontal temporal dementia (FTD) have the worst insight compared to people with Alzheimer's disease (AD) and non‐clinical controls, but, in general, people with dementia underestimate how unassured and submissive they have become, preserving a view of themselves as just as assertive and sociable as they were before the illness (Rankin et al., 2005, p. 636).

To sum up, in this section, we offered some reasons in favour of what we will refer to as the *Psychological Benefits claim*: some CMDs are psychologically beneficial as they are reliably correlated with increased well‐being in people with neuropsychological disorders featuring memory impairment. As a result of the distortions, the person feels better about herself. She might feel more competent than she actually is because she believes she is aware of important events in her life history, or she might feel more self‐assured than she actually is because she believes that she still is as assertive and extroverted as she was before the onset of the disease.

## 5 EPISTEMIC INNOCENCE AND CLINICAL MEMORY DISTORTIONS

In Sections 3 and 4, we offered some reasons to endorse the *Epistemic Costs claim* and the *Psychological Benefits claim* for at least some CMDs. In some contexts, such as in the debate on the SPECAL method, it has been suggested that it is *because* memory distorts reality that the psychological benefits ensue.^2^ The idea is that some autobiographical memories increase well‐being *by* distorting reality. This may be true, but we shall argue that, often via their psychological benefits, some CMDs also have epistemic benefits. In particular, they contribute to restoring epistemic functionality by preventing serious epistemic harms from occurring at a critical time.

Thus, we defend an *Epistemic Benefit* claim regarding CMDs and invoke the notion of *epistemic innocence* to describe CMDs. There are two senses of ‘innocent’ that resonate with the use we propose, and both come from the legal account of innocence defence (see Botterell, 2009). In *justification defence*, an act that is objectionable is not condemned because it prevents serious harm from occurring. Innocence here is due to the act being an effective response to an emergency situation. In legal contexts, self‐defence is the most common example of this form of innocence. The person is not criminally liable for acting in self‐defence even though her act would in other circumstances constitute an offence. In *excuse defence,* an act that is objectionable is not condemned because the person performing it either could not have done otherwise (e.g., as in duress or compulsion) or did not realise that the act was objectionable (e.g., due to intoxication or insanity). Innocence here is due to the person not being responsible for performing the act.^3^ It is useful to apply the notion of innocence defence to the epistemic domain. We can think about whether adopting a belief that is inaccurate or ill‐grounded has any epistemic benefits (see Bortolotti, 2015, 2016; Sullivan‐Bissett, 2015), and we can ask whether a person is responsible or blameworthy for having such a belief (see Bortolotti & Miyazono, 2016). Having a distorted memory is epistemically innocent if and only if:

(*Epistemic Benefit*) the memory delivers some significant epistemic benefit to an agent, such as the prevention of a serious epistemic harm; and

(*No Alternatives*) a less distorted memory that would deliver the same epistemic benefit is not available to that agent.

A distorted memory can be innocent in the sense of justification defence by preventing epistemically significant harms (such as the loss of the sense of self). A distorted memory can be innocent in the sense of excuse defence if a more accurate memory is not available due to a cognitive impairment. The clinical context in which the distortion is produced is important to the innocence claims: non‐clinical memory distortions may not prevent epistemic harms to the same extent as clinical ones and may be corrected more easily in the absence of cognitive impairments.

So far, we presented the framework of epistemic innocence, according to which epistemically costly cognitions can also be epistemically beneficial. Next, we will argue that some CMDs are epistemically innocent.

### 5.1 The *Epistemic Benefit* condition

Here, we focus on two main types of epistemic benefits, deriving from increased socialisation and the retention of key beliefs about the self.

*Increased socialisation*. There are epistemic and psychological pay‐offs to having CMDs as opposed to not having autobiographical memories to report or having CMDs with motivationally enhanced content as opposed to autobiographical memories that are more realistic. Some autobiographical information to report as opposed to none enables people to exchange information with peers and receive feedback from them. It has been shown that even a small amount of social interaction can significantly improve memory and cognitive performance (Ybarra et al., 2008). Moreover, people whose CMDs have motivationally enhanced content feel better about themselves, feeling more competent and confident and managing more effectively the negative emotions associated with their illness and their memory loss.

In addition to threatening socialisation, stress and anxiety further compromise epistemic functionality by reducing attention and concentration. As we saw, when the CMD is motivationally biased, it contributes to the construction of a better self or a better reality (e.g., ‘I was an excellent football player when I was younger’, ‘I am still as confident and as easy‐going as before I started having memory problems’). Such beliefs are instrumental to managing negative feelings that could become overwhelming. The defensive role of memory distortions and their positive effects on the person's happiness or well‐being has been observed before (McKay & Dennett, 2009), but here, we suggest that the successful management of negative emotions in people with CMDs is likely to have some significant epistemic benefits that have been overlooked. Free from the extreme stress that they would suffer if they had more realistic beliefs about themselves and their disease, people are more likely to interact with the surrounding environment in a way that is conducive to acquiring new information and receiving feedback via meaningful social exchanges (Bortolotti, 2016).

In dementia, for instance, people lose the capacity to remember events in the remote past and to form new memories. This compromises their communication skills, thereby affecting their capacity and willingness to exchange information with other people and receive feedback from them (Small, Geldart, Gutman & Clarke Scott, 1998, p. 291). Social isolation has a number of negative effects on mental function more generally, and on the capacity to ‘express and explore identity’ (Bouchard Ryan, Banniser & Anas, 2009, p. 145) more specifically. By filling gaps in one's knowledge about the past and offering a (partially illusory) sense of competence, memory distortions can increase self‐confidence and support the level of communication required for meaningful social interactions. Stories about oneself are a means of expression and communication, and some of their functions are not compromised by local inaccuracies in the reporting. Autobiographical events may be reported with inaccurate time tags or in the wrong chronological order, and, when they are reported repeatedly, details in different versions of the same story may be inconsistent with one another. These features have also been seen as the result of adaptive mechanisms. Retelling events that are representative of the teller's identity ensures that the key event is not forgotten, although some details may be misrepresented. The story remains available to be shared, enabling social exchanges and feedback on it (Hydén & Örulv, 2009, p. 206).

*Preservation of key beliefs about the self*. By having CMDs and reporting them, people also retain some true beliefs about themselves that are supported or implied by the content of the CMD. This is of crucial importance at an advanced stage of dementia, when autobiographical information is increasingly threatened by memory impairments. Even when memories are partially inaccurate, they represent a means by which true beliefs about the self are retained. By having and reporting CMDs, people preserve autobiographical material that can contribute to self‐knowledge.

Fotopoulou identifies an important epistemic role of memory distortions in RM (the person with brain damage who had an inflated conception of past achievements):

RM's relatives confirmed that several of his confabulations were false versions of real past experiences. These had typically been important to RM and may have served as sources of personal identity. (Fotopoulou, 2008, p. 555)

The memory reports we are considering here are distorted in the sense that they are ‘false versions of real past experiences’, as Fotopoulou puts it, and the autobiographical content in those reports is remembered by the person, shared with others, and more likely to be remembered in the future because of its availability for reporting. If loss of autobiographical memory leads to the agent losing her sense of self, the reporting of CMDs counteracts that loss by making available key autobiographical information that is part of one's identity for sharing and for recall. This is an epistemic benefit that is associated with psychological benefits, but is not mediated by them, as it contributes directly to an epistemic goal, that is, the retention of some true beliefs about the self.

In the context of dementia, it is widely accepted that loss of autobiographical memory weakens the sense of self and detracts from self‐knowledge (Della Sala, Freschi, Lucchelli, Muggia & Spinnler, 1996; Orona, 1990). Addis and Tippett (2004) measured the *strength* (number of responses), *quality* (specificity of responses), *complexity* (number of categories sampled in the responses), and *direction* (positivity or negativity) of sense of self in a sample of people with mild‐to‐moderate Alzheimer's disease in comparison to a non‐clinical sample of elderly people. Loss of autobiographical memory was correlated with people generating fewer and more abstract responses to sense of self questions and having a more negative sense of self than controls. In particular, the inability to remember autobiographical events occurring in childhood or early adulthood (*self‐defining memories*) was key to the poor quality in people's sense of self.

Consider the following case. A woman has a vivid recollection of walking on the beach with her parents. She believes the event occurred that morning, but it occurred 60 years earlier, when she was a teenager. Such a distorted memory involves a number of false beliefs (e.g., that the woman's parents are still alive and that she is still young). However, in a context in which access to autobiographical information is limited and declining, as in dementia, such a vivid recollection may help the woman connect with important aspects of her personal history in the absence of other reliable information about her childhood and her relationship with her parents. The memory can help her think of herself as, say, the girl who lived near the sea and loved taking walks on the beach with her parents. Thus, reporting the memory supports the woman's sense of self, despite the fact that it will reveal gaps and inconsistencies in her self‐narrative (e.g., ‘Where are my parents now?’).

Here is another case. In *Keeping Mum*, Talbot (2001, p. 22) describes how her mother was a great storyteller before she had dementia, and one of her best stories was how one day, when she was 14, she was late for school because her mother had just given birth to twins. The school headmistress did not believe that that was the reason for being late and punished her, which she felt was a great injustice. When dementia advanced, the story about the twins’ birth ended up being merged with other stories (for instance, other stories about being late for school) and was repeated many times. Repetitive scripts enabling people to remember key features about themselves (the unfairly punished teenager, the able gardener, the good bridge player) help retain some sense of self and integrate information about their lives before the illness into their current personal narratives (Harman & Clare, 2006, p. 484).

A further example is Martha, suffering from Alzheimer's disease, who often told the story of how she learnt to drive, and she bought her own car, defying the doubts of her husband and her own family (Hydén & Örulv, 2009, p. 207). This was something she was presumably very proud of because not many women at the time did the same. Not only was the story told many times in conversation, but aspects of the story would also be repeated frequently during the same conversation. Notwithstanding repetitions and inconsistencies, the story played a key role in Martha's sense of self by reinforcing the conception of herself as someone who would ‘follow her own mind' and not care about the disapproving attitudes of those closest to her.

CMDs qualify as an emergency response because the benefits conferred by them depend on people finding themselves in an already compromised epistemic state. Their sense of self is significantly weakened by gaps in autobiographical memory and overwhelming negative emotions, with adverse effects for attention and concentration. Both increased socialisation and retention of a sense of self can contribute to epistemic functionality. Epistemic functionality is the capacity to pursue and achieve one's epistemic goals, and it depends on a strong sense of self and an active engagement with one's physical and social environment. As we saw, when there is a loss of autobiographical memory, some of the evidence on which one bases beliefs about the self is no longer available, compromising one's active engagement with the physical and social environment and socialisation. When one is unsure about which person and what type of person one is, one feels incompetent, fears sanction from peers, and withdraws from social interactions.

Thus, CMDs function as *epistemic enabling conditions* and are beneficial insofar as they prevent the occurrence of the epistemic breakdown that could ensue if people experienced an increased loss of socialisation and a weakening of the sense of self. Next, we argue that this result could not be achieved by other, less epistemically costly, means.

### 5.2 The *No Alternatives* condition

To make a case for the epistemic innocence of CMDs, it is not sufficient to point to some epistemic benefits; we also need to rule out that the benefits could be attained in less epistemically costly ways. We will do this by suggesting that having less epistemically costly memories, which would confer the same epistemic benefit, is in some sense *unavailable* to people with neuropsychological disorders featuring memory impairment.

There are different ways in which people may fail to have the relevant cognition (see Sullivan‐Bissett, 2015 for a more detailed description of the taxonomy). The forms of unavailability that are applicable to cases of CMDs are *strict* and *motivational* unavailability, and we consider them in turn.

A cognition is *strictly unavailable* when it is not accessible to the person because it is, for example, opaque to introspection. In the context of memory distortions, an alternative, less epistemically costly memory is impossible to retrieve or reconstruct due to the memory impairments that characterise neuropsychological disorders such as dementia and amnesia. Strict unavailability applies both to alternative cognitions and to the information which would suggest that the reported cognition is distorted or that an alternative cognition is less epistemically costly. For instance, we described the case of a woman with dementia who reports walking on the beach with her parents that very morning when the walk actually occurred 60 years earlier. The woman may not have access to a non‐distorted version of her memory report (a version with the correct time tag) and may also lack access to information that would disqualify her report *as distorted*, such as true beliefs about her age or memories of her parents’ deaths.

A cognition may be *motivationally unavailable* if access to it is strictly speaking possible but inhibited by motivational factors. When the distortion involved can be understood as a defence mechanism, the person denies some unpleasant thing, embellishes, or rationalises it. We have also seen that attending more to positive stimuli, especially with respect to autobiographical information, is at least partly responsible for positive affect despite cognitive decline. There is a sense in which less positively biased reports are motivationally unavailable to the person in this case, perhaps due to the person having some pro‐attitude towards a more positive conception of herself and her past.

Motivational unavailability is weaker than strict unavailability, but in many cases of CMDs, there is an interesting interaction between the two. An appeal to strict unavailability explains why the person does not offer a less distorted, more accurate memory, and an appeal to motivational unavailability explains why the memory distortions she offers tend to have a positively biased content.

Here is an example. We saw that some CMDs fill a gap in memory in the context of a neuropsychological disorder. Gap filling might be pragmatically beneficial insofar as it allows the subject to avoid dumbfounding. A memory distortion in this context prevents the person from having to claim that she does not know something that she is socially expected to know in response to a question about her autobiographical past. Here is another example. We saw that a positivity bias is frequently observed in CMDs and might apply to self‐descriptions. People with neuropsychological disorders often describe themselves in terms which indicate that they are healthier than they really are, as well as exaggerating previous status with respect to their social or financial life. In this case the person has a strong motivation to present herself in a positive light or to enhance her present or past reality.

We have suggested that some CMDs meet the *No Alternatives* condition when there is no less epistemically costly memory report that would bring the same epistemic benefits. This tells us that some CMDs, those that bring epistemic benefits and for which there is no available alternative, are epistemically innocent.

### 5.3 Objections

Here we consider two objections to the application of the *Epistemic Benefit* condition to CMDs and one objection to the application of the *No Alternatives* condition to CMDs.

First, is it true that CMDs increase socialisation? In Section 3, we noted that when a person has a distorted memory and reports it, the distortion may be detected, leading to social sanction and reduced exchange. The person may be seen as an untrustworthy communicator and this impairs socialisation. But in Section 6.1, we argued that when the person has a memory distortion and reports it, the report may help provide material for exchange of information, potentially increasing socialisation. So which one is it? Do CMDs reduce or increase socialisation?

The answer depends on what we take to be the terms of the comparison. The person with dementia is less reliable than she was before the onset of the illness when she reports autobiographical memories due to the cognitive impairments that are typical of her illness. As a result, the person may be less willing to answer questions or volunteer information because, when she says something obviously false, she is sanctioned by her peers for not telling the truth. But having and reporting a memory distortion (which has a kernel of truth in it) may be more advantageous for supporting mutual exchange of information and socialisation than saying nothing at all.

Our position is not that memory distortions are epistemically *good*, or that distorted memory reports are epistemically *justified*. Rather, our point is that CMDs have epistemic benefits as well as costs, so benefits and costs need to be weighed against each other, and the costs may outweigh the benefits. The whole point of developing the epistemic innocence framework is that it does not imply that the benefits outweigh the costs. If a memory distortion is epistemically justified, then it is the *right* way to go, epistemically speaking. Epistemic innocence does not have this implication. Epistemically innocent cognitions remain flawed in important ways but also have benefits that need to be taken into account in the epistemic evaluation of these cognitions.

Thus, socialisation can be both harmed and promoted by the having and reporting of distorted memories. The relationship between distortion and socialisation shows the complexity and context‐dependent nature of the problem we are addressing. Having and reporting a distorted memory can be better than not making any contribution to a conversation on one's autobiographical history when the goal is to increase socialisation as it enables the conversation to continue. But if the distortion is detected and challenged, then the effects of the report may include the speaker losing the trust of others and experiencing reduced socialisation as a result.

In some cases, it is difficult to say whether the epistemic benefits of a cognition outweigh its epistemic costs because it is not clear how different epistemic features should be weighed against one another. That is why we want to leave the question about the benefits outweighing the costs open at this stage and dependent on context. For someone whose memory distortions can be easily corrected, the inaccuracy of the memory report may be too high a price for gaining a stronger sense of self or for benefiting from increased socialisation and opportunities for information exchange and feedback. But for someone with a degenerative disorder, someone whose autobiographical memory is going to progressively fade, the benefits of increased socialisation and a stronger sense of self may well outweigh the costs of inaccuracy.

We move now to the second objection relating to the applicability of the Epistemic Benefit condition to CMDs: why is retaining core beliefs about the self an *epistemic* benefit as opposed to a merely psychological one? The concern is that the sense of self is psychologically important, but it does not have a distinct or a significant epistemic role. Our view is that the distinctly epistemic contribution of CMDs is to enable the retention of true beliefs about the self at a time in which autobiographical information is under threat. This is particularly relevant to disorders in which cognitive impairments become more severe over time, such as dementia.

The retention of any true belief can be described as epistemically advantageous. However, core beliefs about the self play an especially important role in the acquisition and retention of self‐knowledge. As we saw, some psychologists tell us that memories of some life events contribute more significantly than memories of other events to people's knowledge of themselves (such memories are often called ‘self‐defining memories’, typically memories of one's childhood and early adulthood). Losing such memories leads to having more vague and abstract beliefs about what kind of person one is. We could call the beliefs based on self‐defining memories ‘self‐defining beliefs’.

The belief that Jane read Philosophy at university can be self‐defining for her, but the belief that she had broccoli for dinner last night is not likely to be. Self‐defining beliefs are not foundational in the sense that they are less vulnerable to sceptical challenges or that the justification of other beliefs necessarily depends on them, but they are special in that they are more central to one's knowledge of the self in terms of continuity (Jane is just the same person who read Philosophy at university) and narrative coherence (Jane is someone who loves Philosophy) than other beliefs about the self. This means that being unable to form self‐defining beliefs is a greater epistemic loss for the prospects of gaining self‐knowledge than being unable to form non‐self‐defining beliefs. As we saw, the retelling of events that are representative of one's key traits, even when the stories are partially inaccurate, helps retain self‐defining beliefs.

One concern about our argument in Section 5.2, concerning the applicability of the *No Alternatives condition,* might be that it is not enough to show that there is no alternative cognition available to the person experiencing clinical memory distortions. One might object that our overall argument requires the claim that the person cannot attain the epistemic benefits we identify by other means, including therapeutic interventions aimed at aiding autobiographical memory recall. If those interventions were available, could they not provide the relevant epistemic benefits, at a lower epistemic cost? This is a point that deserves to be explored further with respect to mental health conditions where loss of autobiographical memory is temporary or can be overcome using therapy. However, in dementia, which is a degenerative condition, we know of no interventions with the relevant epistemic benefits. Should such interventions become available, this would of course be taken into account in an assessment of the epistemic innocence of CMDs.

In this section we defended our position on the epistemic innocence of CMDs from three objections. Next, we discuss its implications.

## 6 IMPLICATIONS OF EPISTEMIC INNOCENCE

We have argued that at least some CMDs are epistemically innocent. As epistemic innocence is a weaker notion than epistemic justification, we are not committed to the claim that the epistemic benefits of CMDs outweigh their epistemic costs. One may wonder, then, whether our claim has any practical implications at all.

Acknowledging the epistemic innocence of at least some memory distortions can make a difference to the management of memory distortions in a clinical context and to the terms of the carer–user relationship where the user has cognitive impairments resulting in distorted memories. Should CMDs be challenged and, if so, in what way?

In some circumstances, there are psychological reasons not to directly confront people who make such reports, and these have been discussed in the recent psychological literature. Confronting people about their CMDs might cause them anxiety and distress and make them lose the self‐confidence necessary for social exchanges. There has been a lively debate about how one should relate and react to people who make false reports when they do so without the intention to deceive and in the context of serious cognitive impairments. Some have convincingly argued that providing direct feedback and confronting people with neuropsychological disorders, especially when they have not had an opportunity to adjust to the effects of memory loss, can be detrimental and increase distress (Fotopoulou, 2008, p. 546). Fotopoulou suggests adopting a non‐confrontational attitude when treating people who confabulate, and referring to RM (the person with brain damage who had an inflated conception of past achievements), she writes:

[C]linicians could try to explore with patients and their relatives the subjective meaning of these confabulations for the continuity of patients’ self‐identity and the preservation of self‐esteem. For example, for as long as RM's confabulations did not directly impede his everyday activities, responsibilities and social interactions, rehabilitation staff were encouraged to refrain from directly confirming or contradicting RM's confabulations. Instead, they were asked (1) to respond to his statements at face value with natural interest and curiosity, (2) to discreetly suggest and add correct background information to his stories, when possible, (3) to pace the conversation and help RM to stay within a given conversational topic, and (4) to explore memories and current facts in ways that take into account both his emotions and also the emotions of others and their need for a shared reality. (Fotopoulou, 2008, p. 556)

In *Contented Dementia*, as we saw earlier, James argues that caregivers should not challenge but actively encourage people with Alzheimer's disease and possibly other forms of dementia to have memories and beliefs that can be partially inaccurate. James maintains that, despite factual inaccuracies, delusional beliefs and distorted memories are instrumental to the person regaining self‐confidence and retaining meaningful interactions with those around them. For instance, some of the beliefs and memories to be encouraged according to James are those contributing to key aspects of a person's sense of self: people with dementia may present themselves as the able gardener or the good bridge player, remembering some past achievements and falsely believing that the relevant skills have been preserved. The main contention of the book is that the caregiver should be supportive and agree with most of what the person with dementia says in order to minimise stress, maximise well‐being, and build a working interpersonal relationship which brings mutual contentment. Such arguments derive from psychological considerations and aim to enhance the well‐being of the person with cognitive impairments.

But if CMDs were tolerated or encouraged, the worry is that the person with cognitive impairments would find herself in situations where her epistemic standing is compromised and, moreover, in situations that would lack authenticity because the people around her would allow her to have an inaccurate representation of reality in order to prevent her from becoming distressed. That is why the SPECAL method proposed by James is still very controversial. Even if it were successful in achieving the goal it sets itself, that is, making the life of people with Alzheimer's disease less distressing and more pleasant, there is some predictable resistance to adopting such a method because it asks caregivers to deceive. They are trading *epistemic goods* for *psychological ones* on behalf of the people they care for.

Such worries are understandable, but the relationship between the psychological and epistemic effects of CMDs is more complex than the debate on the SPECAL method suggests. We argued that the psychological benefits of CMDs can lead to epistemic benefits by supporting the person's previously compromised epistemic functionality and also that some CMDs have independent epistemic benefits, not mediated by psychological ones, when they allow the person to retain some true beliefs about herself in situations where the retention of such beliefs is threatened by memory loss.

Whether CMDs should be challenged cannot be settled only by thinking about whether doing so would increase or decrease distress. If CMDs also have consequences that are epistemically relevant, those should be taken into account. If CMDs have epistemic benefits, then the clinician or carer who avoids challenging the user may serve not only her psychological interests but also her epistemic ones. The epistemic benefits of CMDs provide additional reasons not to confront people about their inaccurate memory reports because CMDs support the person's epistemic functionality and enable her to retain some key autobiographical information. Critically, confronting the person about the distorted memory report may not enable her to correct the distortion given that a less epistemically costly memory may not be available.

Our discussion of the epistemic benefits of memory distortions does not amount to a vindication of any specific approach to caring for people with dementia, and it does not follow from it that CMDs should always go unchallenged. Rather, the suggestion is that we should evaluate the role of CMDs in the overall cognitive economy of the relevant clinical population and of the individual and allow clinical interventions and interpersonal regulation to be informed not just by the psychological effects of CMDs but also by their epistemic effects. If distorted memories enable the person to react positively to the setbacks caused by the effects of illness on her life and to engage in effective communication, or to retain some self‐defining beliefs, then there would be reasons for not challenging those distorted memories for as long as they deliver the identified benefits.

## 7 CONCLUSIONS

In this article, we discussed the effects of CMDs, focusing on their potential epistemic benefits. Our target here was memory reports that misrepresent reality but are not offered with an intention to deceive. We briefly summed up the epistemic costs of memory distortions, which included their failing to accurately represent reality, their content being vague and abstract relative to accurate memories, their being in tension with the person's other beliefs, and their hindering the communicator's perceived trustworthiness. We then reviewed some psychological benefits of memory distortions. These benefits included preserving a competent and coherent sense of self and creating better selves, thereby managing negative emotions and increasing well‐being and socialisation.

Next, we appealed to the framework of *epistemic innocence,* a notion intended to capture those cognitions that are epistemically costly but also have significant epistemic benefits that are not otherwise attainable. We applied the notion of epistemic innocence to the case of CMDs. We argued that having CMDs is epistemically beneficial insofar as they can support epistemic functionality and help retain some true beliefs about the self. We also argued that, in some cases, such epistemic benefits would not be attainable without the memory distortion because alternative, less epistemically costly memories are often unavailable to the person in the context of neuropsychological disorders featuring memory impairments.

Finally, we turned to the implications of our thesis for symptom management in clinical practice and for the carer–user relationship. On the basis of the idea that some CMDs are epistemically innocent, we argued that decisions about whether, how, and when some symptoms of neuropsychological disorders featuring memory impairments are challenged should be informed not only by considerations about the person's well‐being but also by considerations about her epistemic situation. Just as there are good reasons not to challenge CMDs from a psychological point of view, there may be good reasons not to challenge them from an epistemic point of view.

We have not claimed that the epistemic benefits of CMDs always outweigh their epistemic costs, or even that all CMDs have epistemic benefits that are significant. Rather, we observed that the potential epistemic benefits of CMDs have been neglected in the recent debates, and we have hinted at what the consequences of acknowledging those benefits might be. More evidence is needed to support the claim that CMDs are epistemically beneficial in the way we suggested, and we hope that philosophers’ interest in this area will create the opportunity for empirical studies aimed at testing and refining the claim.

## Footnotes

1 Of course, being married to a person for only 4 months *and* having four children with that person is not inconsistent; it might just be that the marriage came later on in the relationship. The supposed inconsistency here results from the cultural context in which the confabulation was offered.

2 The idea that memory has distinct functions that may pull in different directions is not new. For instance, some have argued that remembering is at the same time an act of *veracity* and an act of *utility* (Neisser & Fivush, 1994). Memories connect people to the past and thus contribute to knowledge but are also re‐interpreted to provide a sense of continuity and direction to people's life stories and to match people's present goals and concerns (Conway & Loveday, 2015; Fernández, 2015). The aims of veracity and utility may conflict, and the utility of some memory reports may depend on their foregoing veracity.

3 According to Botterell (2009), the distinction between the two senses of innocence is controversial in the legal literature. Those who believe that there is a genuine distinction between the two talk about the person's responsibility for the action or about her entitlement to act. In one version of the distinction, a justification seems to provide a stronger defence than an excuse: the person whose act is justified was entitled to act in that way, whereas the person whose act is excused is merely not blameworthy for performing it. The distinction might be understood in an analogous way in the epistemic context, although we do not discuss that here.
